# Association between nighttime sleep duration, nap time, and mild cognitive impairment in Chinese older adults: a cross-sectional study

**DOI:** 10.1186/s12889-024-19900-0

**Published:** 2024-09-02

**Authors:** Yanliqing Song, Haoqiang Liu, KeNan Gu, Yue Liu

**Affiliations:** 1 College of Sports, Nanjing Tech University, Nanjing, China; 2 School of Athletic Performance, Shanghai University of Sport, Shanghai, China

**Keywords:** Nighttime sleep duration, Nap time, Health promotion, CHARLS, Mild cognitive impairment

## Abstract

**Objective:**

This study aims to investigate the relationship between midday nap time, nighttime sleep duration, and mild cognitive impairment (MCI) in Chinese older adults and determine the recommended sleep duration to provide a scientific basis for preventing and managing MCI in this population.

**Methods:**

Utilizing the 2020 China Health and Retirement Longitudinal Study database, the demographic data, health status, and lifestyle information of the study participants were collected. A total of 5,314 valid samples were included in the analysis. Logistic regression and restricted cubic spline plots were employed to explore the relationship between sleep patterns and MCI.

**Results:**

In the cross-sectional analysis, a linear relationship was observed between midday nap duration and MCI among Chinese elderly. The probability of MCI was lowest among those who napped for less than 30 min at noon. Compared with individuals who napped for30-90 min, those who did not nap were more likely to have MCI (OR = 1.30, 95% CI: 1.05–1.60). Older adults with napping duration < 30 min (OR = 0.73, 95% CI:0.56–0.95) also exhibited lower probability of MCI when compared those without napping habit, Nighttime sleep duration exhibited a U-shaped relationship with MCI. Individuals with less than approximately 6 h of nighttime sleep showed a gradual decrease in the probability of MCI with increasing sleep duration, whereas those with more than 7.5 h demonstrated an increase in the probability of MCI with longer sleep duration. Older adults who slept less than 6 h at night (OR = 1.22, 95% CI: 1.01 ~ 1.46) or more than 8 h (OR = 1.78, 95% CI: 1.35–2.33) were more likely to develop MCI compared with those who slept 6 to 8 h.

**Conclusion:**

After controlling for potential confounding variables, both nighttime sleep duration and midday nap duration exhibited a nonlinear “U”-shaped relationship with MCI among the elderly. The probability of depression was lower with a nap duration of approximately 60 min, and the optimal nighttime sleep duration was 6–8 h, with around 7 h providing the greatest cognitive benefits.

## Introduction

Against the backdrop of the accelerated aging of the population, cognitive impairment has emerged as a major public health concern demanding immediate attention. Impaired cognitive function not only results in functional dependence [1]and diminished health-related quality of life [2] but also imposes a substantial burden on caregivers [3], thereby creating a societal challenge [4]. Hence, the factors influencing the cognitive decline process must be identified to safeguard and preserve cognitive function in the elderly population.

Existing literature suggests a correlation between sleep duration and cognitive function [5, 6], with shorter sleep durations typically associated with higher cortical β-amyloid burden, a precursor to cognitive decline [7]. Reports indicate an estimated 15.07 million cases of dementia among the elderly in China, including 9.83 million patients with AD dementia and 38.77 million with mild cognitive impairment (MCI) [8]. MCI represents a transitional phase between normal aging and AD and is often viewed as a prodromal stage of AD. Numerous studies have demonstrated a strong relationship between sleep and cognitive function [9, 10]. Sleep deprivation has been linked to increased concentrations of β-amyloid in the brain and elevated levels of inflammatory factors [11]. It also alters hypothalamic–pituitary–adrenal axis activity, culminating in cognitive dysfunction [12]. Conversely, prolonged periods of sleep may accelerate the rate of frontotemporal gray matter atrophy in older adults, potentially impairing memory and precipitating cognitive dysfunction. Therefore, proactive sleep intervention holds promise as a novel approach for the clinical prevention and treatment of AD, emphasizing the critical role of sleep management in preserving cognitive health among the elderly population. Previous research has demonstrated that most people recognize sleep disorders as a risk factor for dementia and exhibit a positive attitude towards improving sleep quality and acquiring knowledge about dementia. Additionally, a positive correlation has been found between people’s knowledge and their positive attitudes, indicating the importance of acquiring knowledge about improving sleep disorders for future dementia prevention [13].

Sleep time typically refers to the cumulative amount of sleep acquired within a 24-hour period, including nighttime sleep and daytime naps. Although previous research on sleep duration has primarily focused on nighttime sleep [14], the importance of daytime naps in sleep patterns should be acknowledged [15]. Globally, 22–69% of the elderly population engage in napping habits, with the prevalence among the Chinese older adults reaching as high as 68.6% [16]. Previous studies have combined nighttime sleep and daytime nap duration into total sleep time when investigating MCI in Chinese older adults, often overlooking the specific relationship between daytime napping and MCI [17]. Hence, this study examines the relationship between nap time, nighttime sleep duration, and MCI among Chinese older adults, aiming to delineate the recommended sleep duration. By doing so, the study endeavors to offer a scientific and rational foundation for the prevention and management of MCI in the elderly.

## Methods

### Study population

The data utilized in this study are sourced from the 2020 CHARLS database. CHARLS is the leading nationally representative survey of the middle-aged and elderly population in China, employing a multi-stage PPS random sampling method grounded in implicit stratification to ensure comprehensive sample representation [18]. The CHARLS questionnaire encompasses various modules, including sociodemographic data, psychological status, and health status, providing robust data for exploring the relationship between nap duration, nighttime sleep duration, and MCI in Chinese older adults and determining optimal sleep durations. The ethical approval for the 2020 CHARLS survey was granted by the Biomedical Ethics Committee of Peking University under approval number IRB00001052-11015. During the field survey, each consenting respondent was required to sign two informed consent forms, with one copy retained by the respondent and the other deposited in the CHARLS office. The study’s inclusion criteria stipulated older adults aged 60 years and older. Initially, 7,880 elderly individuals were recruited for the study. Missing samples of demographic variables and Epidemiological Center Depression Scale scores were excluded from the analysis. After applying the inclusion and exclusion criteria, a total of 2,566 participants were excluded. The reasons for exclusion included incomplete data, failure to meet the inclusion criteria, and withdrawal from the study. Consequently, the final effective sample comprised 5,314 participants.

### Main variables

#### Mild cognitive impairment

The cognitive function assessment in the CHARLS survey follows the methodology utilized in the American Health and Retirement Study (HRS) [19]. Participants underwent face-to-face evaluations across four dimensions of cognitive functioning: orientation, memory, calculation, and drawing. For orientation and calculation, the Cognitive State Telephone Interview was employed. Orientation was assessed by querying participants about the year, month, day, day of the week, and current season. Each correct response earns 1 point, resulting in a total score of 5 points for this dimension. Calculation ability was evaluated by asking participants to subtract 7 from 100 consecutively, repeating this process five times. Each correct calculation earns 1 point, yielding a total score of 5 points. Memory assessment involved the immediate recall of 10 randomly presented words. Participants were tasked with recalling as many words as possible immediately following presentation. Additionally, delayed recall was assessed after participants finished the depression scale survey, calculation, and drawing tests. The total memory score is the sum of points earned for immediate and delayed word recall, with each correctly recalled word worth 1 point. Thus, the maximum score for memory is 20 points. Drawing ability was evaluated by presenting participants with a picture and assessing their capacity to reproduce it accurately. Correct reproduction earns 1 point. The total cognitive score was calculated as the sum of scores from all four dimensions: orientation (5 points), calculation (5 points), memory (20 points), and drawing (1 point), resulting in a maximum score of 31 points [20].

Without consensus on the diagnostic criteria for MCI, we defined MCI on the basis of aging-related cognitive decline (AACD) in our study [21]. Specifically, individuals were classified as having MCI if their cognitive performance fell at least 1 standard deviation (SD) below age-specific norms. Participants aged 60 years and older were categorized into groups spanning every five years. Within each age group, individuals whose cognitive performance met the AACD criteria (i.e., scoring at least 1 SD below age-specific norms) were identified as having MCI.

#### Sleep duration

Nighttime sleep duration was assessed using the question: How many hours did you typically sleep each night in the past month? In a meta-analysis of 35 sleep studies, participants were categorized into three groups based on sleep duration: short sleep (< 6 h), moderate sleep (6–8 h), and long sleep (> 8 h) [22]. Nap duration was evaluated using the question: How much sleep did you typically get during daytime naps in the past month? Participants were segmented into four groups: no nap (0 min), short nap (< 30 min), medium nap (30–90 min), and long nap (> 90 min). The selection of these time cut-off points was informed by prior epidemiological literature on daytime napping [16].

### Covariates

For this study’s design requirements, sociodemographic data, health status, and other indices were extracted from the 2020 CHARLS database as independent variables. The variables across different dimensions were assessed on the basis of their types, with categorical variables assigned values accordingly. For binary categorical variables, a value of 0 was assigned for one category and 1 for the other. For categorical variables with three or more categories, values were assigned in increments (e.g., 0, 0.5, 1, and so on) to represent each category. The specific variables selected for evaluation are as follows:

1. Sociodemographic data: age, gender, education, region, occupation, marital status, and living arrangement;
2. Health status: individual comorbidities, self-rated health, frequency of physical exercise per week, smoking, and alcohol consumption.

### Statistical analysis

Statistical analysis was conducted using R4.2 software. Continuous variable characteristics were described using Kolmogorov–Smirnov tests to assess normal distribution. Normally distributed data were presented as (x ± s), with t-tests for between-group comparisons and analysis of variance for multiple group comparisons. Non-normally distributed data were described as M(Q1,Q3), with rank-sum tests for group comparisons. Categorical data were expressed as n (%) and compared using the chi-square test or Fisher’s exact test. Multicollinearity among independent variables was assessed using the variance inflation factor (VIF). The Box–Tidwell method tested the linear relationship between logarithmic variables. Logistic regression models were constructed with nap time and nighttime sleep duration as reference groups. The first model included only nap time and nighttime sleep duration. The second model incorporated significant demographic variables identified in univariate analysis. The third model encompassed all significant univariate analysis variables. A restricted cubic spline plot explored the dose-response relationship between sleep duration and depression among the elderly. A significance level of *p* < 0.05 was utilized for two-sided tests.

## Results

### Participant characteristics

The effective sample was 5314 elderly people, including 2899 females, accounting for 54. 55%, the average age of the elderly was (67.50 ± 5.83) years, 749 (14.1%) had MCI symptoms. Table 1 presents the characteristics of the subjects. Among the nighttime sleep groups, the proportion of elderly individuals with MCI was lowest in the group with 6–8 h of nighttime sleep, compared to other groups. The proportion of elderly individuals with MCI was highest in the group with more than 8 h of nighttime sleep. Among the nap sleep groups, the proportion of elderly individuals with MCI was lowest in the group with less than 30 min of nap sleep, while the proportion was highest in the group with no nap habit.

**Table 1:** Basic characteristics of study participants(*n* = 5314)

Variable	All (*n* = 5314)	Nighttime sleep			*P*	Nap sleep time				*P*
		< 6 h (*n* = 2268)	6–8 h (*n* = 2565)	>8 h (*n* = 481)		0 min (*n* = 1957)	<30 min (*n* = 775)	30–90 min (*n* = 1465)	>90 min (*n* = 1117)	
Gender, n (%)					< 0.001					< 0.001
male	2415 (45.45)	858 (37.83)	1321 (51.50)	236 (49.06)		767 (39.19)	292 (37.68)	730 (49.83)	626 (56.04)	
Region, n (%)					0.039					0.005
countryside	4805 (90.42)	2066 (91.09)	2294 (89.43)	445 (92.52)		4805 (90.42)	1798 (91.88)	681 (87.87)	1310 (89.42)	
Marital status, n (%)					< 0.001					< 0.001
married	4132 (77.76)	1683 (74.21)	2083 (81.21)	366 (76.09)		4132 (77.76)	1456 (74.40)	609 (78.58)	1173 (80.07)	
divorce	54 (1.02)	28 (1.23)	23 (0.90)	3 (0.62)		54 (1.02)	28 (1.43)	6 (0.77)	13 (0.89)	
widowed	1101 (20.72)	548 (24.16)	444 (17.31)	109 (22.66)		1101 (20.72)	458 (23.40)	158 (20.39)	276 (18.84)	
unmarried	27 (0.51)	9 (0.40)	15 (0.58)	3 (0.62)		27 (0.51)	15 (0.77)	2 (0.26)	3 (0.20)	
Living alone, n(%)					< 0.001					0.007
yes	498 (9.37)	251 (11.07)	199 (7.76)	48 (9.98)		498 (9.37)	216 (11.04)	57 (7.35)	122 (8.33)	
Health self-assessment, n (%)					< 0.001					0.026
very bad	458 (8.62)	275 (12.13)	149 (5.81)	34 (7.07)		458 (8.62)	196 (10.02)	81 (10.45)	103 (7.03)	
bad	1196 (22.51)	610 (26.90)	484 (18.87)	102 (21.21)		1196 (22.51)	453 (23.15)	157 (20.26)	343 (23.41)	
fair	2607 (49.06)	1054 (46.47)	1334 (52.01)	219 (45.53)		2607 (49.06)	912 (46.60)	387 (49.94)	730 (49.83)	
good	522 (9.82)	175 (7.72)	289 (11.27)	58 (12.06)		522 (9.82)	197 (10.07)	72 (9.29)	147 (10.03)	
very good	531 (9.99)	154 (6.79)	309 (12.05)	68 (14.14)		531 (9.99)	199 (10.17)	78 (10.06)	142 (9.69)	
Smoking n (%)					< 0.001					< 0.001
yes	2144 (40.35)	789 (34.79)	1141 (44.48)	214 (44.49)		701 (35.82)	273 (35.23)	645 (44.03)	525 (47.00)	
Drinking, n (%)					< 0.001					< 0.001
yes	1677 (31.56)	650 (28.66)	889 (34.66)	138 (28.69)		545 (27.85)	232 (29.94)	469 (32.01)	431 (38.59)	
Level of education, n (%)					< 0.001					< 0.001
illiterate	3198 (60.18)	1487 (65.56)	1402 (54.66)	309 (64.24)		1260 (64.38)	490 (63.23)	822 (56.11)	626 (56.04)	
elementary school	1201 (22.60)	469 (20.68)	626 (24.41)	106 (22.04)		401 (20.49)	169 (21.81)	348 (23.75)	283 (25.34)	
Junior high school and above	915 (17.22)	312 (13.76)	537 (20.94)	66 (13.72)		296 (15.13)	116 (14.97)	295 (20.14)	208 (18.62)	
Frequency of physical activity, n(%)					0.179					0.009
0 times a week	670 (12.61)	299 (13.18)	300 (11.70)	71 (14.76)		261 (13.34)	75 (9.68)	169 (11.54)	165 (14.77)	
1–4 times a week	765 (14.40)	340 (14.99)	358 (13.96)	67 (13.93)		270 (13.80)	104 (13.42)	232 (15.84)	159 (14.23)	
More than 4 times a week	3879 (73.00)	1629 (71.83)	1907 (74.35)	343 (71.31)		1426 (72.87)	596 (76.90)	1064 (72.63)	793 (70.99)	
Age group, n (%)					< 0.001					< 0.001
60–69 years old	3492 (65.71)	1418 (62.52)	1797 (70.06)	277 (57.59)		3492 (65.71)	1271 (64.95)	551 (71.10)	982 (67.03)	
70–79 years old	1577 (29.68)	722 (31.83)	679 (26.47)	176 (36.59)		1577 (29.68)	582 (29.74)	194 (25.03)	423 (28.87)	
80 years of age and above	245 (4.61)	128 (5.64)	89 (3.47)	28 (5.82)		245 (4.61)	104 (5.31)	30 (3.87)	60 (4.10)	
Occupation, n (%)					< 0.001					0.544
retire	42 (0.79)	14 (0.62)	27 (1.05)	1 (0.21)		42 (0.79)	11 (0.56)	7 (0.90)	16 (1.09)	
Work after retirement	81 (1.52)	25 (1.10)	49 (1.91)	7 (1.46)		81 (1.52)	25 (1.28)	14 (1.81)	23 (1.57)	
employed	3547 (66.75)	1440 (63.49)	1800 (70.18)	307 (63.83)		3547 (66.75)	1338 (68.37)	519 (66.97)	958 (65.39)	
Unemployed	1644 (30.94)	789 (34.79)	689 (26.86)	166 (34.51)		1644 (30.94)	583 (29.79)	235 (30.32)	468 (31.95)	
Individual comorbidities					< 0.001					0.177
Yes	3978 (74.86)	1833 (80.82)	1782 (69.47)	363 (75.47)		1436 (73.38)	598 (77.16)	1108 (75.63)	836 (74.84)	
MCI, n (%)					< 0.001					< 0.001
yes	749 (14.09)	368 (16.23)	279 (10.88)	102 (21.21)		331 (16.91)	90 (11.61)	177 (12.08)	151 (13.52)	

### Logistic regression of the relationship between sleep duration and MCI in older adults

Multicollinearity diagnosis revealed that all VIF values in our data were below 10, indicating no multicollinearity concerns. The Box–Tidwell test confirmed a linear relationship between continuous independent variables (age, nighttime sleep, nap time) and the dependent variable (MCI) (*P* > 0.05). Logistic regression analysis, controlling for all covariates in model 3, demonstrated that 6–8 h of nighttime sleep served as a protective factor against MCI, whereas more than 8 h of nighttime sleep increased the probability of MCI compared with older adults sleeping < 6 h at night. Additionally, < 30 min and 30 to 90 min of nap time were protective against MCI compared with older adults without napping habits. A nap duration of 90 min showed no association with MCI in older adults (Table 2).

**Table 2:** Logistic regression table of the relationship between sleep duration and MCI in the elderly

Variable	Model one	Model two	Model three
Nighttime sleep, n (%)			
< 6 h	1.00 (Reference)	1.00 (Reference)	1.00 (Reference)
6–8 h	0.65 (0.55 ~ 0.77) **	0.82 (0.69 ~ 0.98) *	0.82 (0.69 ~ 0.99) *
>8 h	1.43 (1.11 ~ 1.83) **	1.48 (1.14 ~ 1.93)*	1.46 (1.12 ~ 1.91)*
Nap sleep time, n (%)			
0 min	1.00 (Reference)	1.00 (Reference)	1.00 (Reference)
<30 min	0.66 (0.51 ~ 0.85) **	0.71 (0.54 ~ 0.92)*	0.73 (0.56 ~ 0.95) *
30–90 min	0.71 (0.58 ~ 0.86) **	0.80 (0.65 ~ 0.98) *	0.80 (0.65 ~ 0.99)*
>90 min	0.77 (0.63 ~ 0.96) *	0.88 (0.70 ~ 1.10)	0.88 (0.70 ~ 1.10)
Nighttime sleep, n (%)			
6–8 h	1.00 (Reference)	1.00 (Reference)	1.00 (Reference)
< 6 h	1.54 (1.30 ~ 1.82) **	1.21 (1.02 ~ 1.45) **	1.22 (1.01 ~ 1.46) *
>8 h	2.19 (1.70 ~ 2.82) **	1.80 (1.38 ~ 2.35) *	1.78 (1.35 ~ 2.33) **
Nap sleep time, n (%)			
30–90 min	1.00 (Reference)	1.00 (Reference)	1.00 (Reference)
0 min	1.48 (1.22 ~ 1.80) **	1.31 (1.07 ~ 1.62) *	1.30 (1.05 ~ 1.60) *
<30 min	0.96 (0.73 ~ 1.25)	0.91 (0.69 ~ 1.21)	0.93 (0.70 ~ 1.24)
>90 min	1.14 (0.90 ~ 1.44)	1.15 (0.90 ~ 1.47)	1.13 (0.88 ~ 1.45)

Note: **Description *P* < 0.01, *Description *P* < 0.05; model 1 includes nighttime and nap time of the elderly, model 2 controls for demographic variables, and model 3 has all covariates

### Dose–response relationship between sleep duration and MCI in older adults

**Fig. 1:**
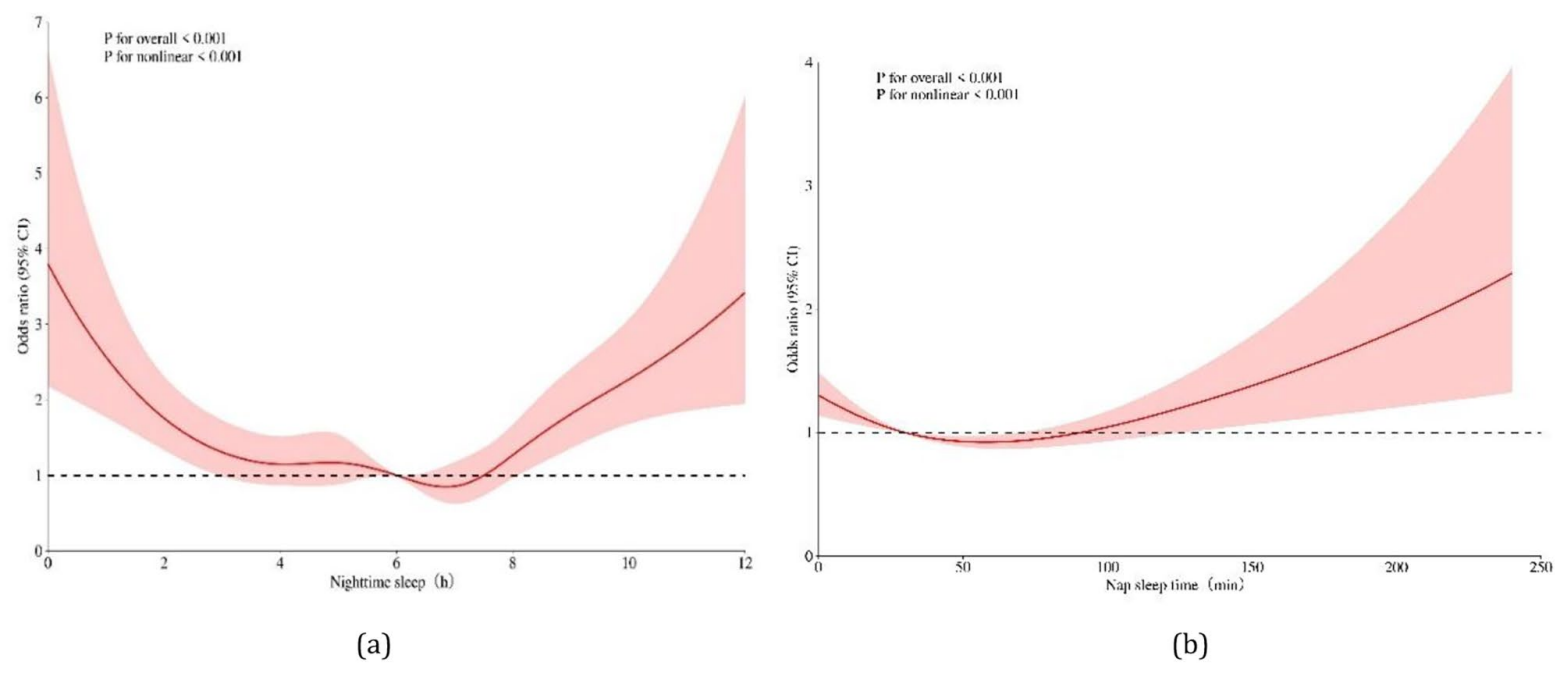
Dose-response relationship between sleep duration and MCI in the elderly. The x-axis represents sleep duration. The y-axis indicates the OR-value calculated by the model. The shadowed area represents the 95% confidence interval (test for overall trend: *P* < 0.001; test for nonlinear trend: *P* < 0.001). In Fig. 1(**a**), the OR gradually decreased with increasing nighttime sleep within the range of sleep time less than7 hours. After surpassing 7 h, the OR gradually increased with increasing sleep time. In Fig. 1(**b**), the OR gradually decreased with increasing nap time within the range of nap time less than 60 min. After surpassing 60 min,, the OR gradually increased with increasing nap time. The red line represents the fitted logistic regression model, and the red area depicts the 95% CI of the fitted curve

The dose–response relationship between sleep duration and MCI among the elderly exhibited a U-shaped curve (P for overall < 0.001; P for nonlinearity < 0.001, and the likelihood of MCI in the elderly decreased from 6 h of sleep to approximately 7 h. In addition, the probability of MCI increased after more than 7.5 h of sleep (Fig. 1a). Given the lowest probability of MCI among the elderly occurred with 6–8 h of nighttime sleep, a logistic regression model was constructed with 6–8 h as the reference group (Table 2). Compared with those sleeping 6–8 h at night, older adults with < 6 h or > 8 h of nighttime sleep were more likely to develop MCI. Regarding nap duration, Nonlinear relationship was observed between nap duration and MCI among the elderly (P for overall < 0.001; P for nonlinear < 0.001) (Fig. 1b). The probability of MCI decreased with increasing nap duration. Considering that the lowest probability of MCI occurred with 30–90 min of nap duration, a logistic regression model was constructed with 30–90 min as the reference group (Table 2). Compared with older adults with 30–90 min of nap duration, those without a napping habit exhibited a higher likelihood of MCI. The duration of nap time in the elderly also exhibits a U-shaped relationship with MCI. As nap time increases, the likelihood of developing MCI continues to decrease, reaching its lowest point at approximately 60 min.

### Discussion

To the best of our knowledge, this study represents the first exploration utilizing a vast, nationally representative longitudinal dataset to investigate the association between nap duration, nighttime sleep duration, and MCI among Chinese elderly, aiming to establish optimal sleep durations. Previous studies have commonly combined sleep durations, potentially obscuring nuanced trends in cognitive decline among the elderly [23]. However, this study employed the RCS method to elucidate potential dose–response relationships between sleep durations and MCI, offering a granular understanding of these associations.

### The nighttime sleep duration of the elderly was U-shaped with MCI

The evidence regarding the association between sleep duration and cognition has been characterized by limited and inconsistent findings. Some studies have reported significantly lower cognitive levels in older adults with both < 6 h and > 9 h of sleep [24], whereas others have found no association between sleep duration and cognition [25]. However, our study revealed a U-shaped relationship between sleep duration and the likelihood of MCI. Meta-analyses have supported this notion, indicating that both shorter and longer sleep durations increase the risk of cognitive dysfunction [22]. Additionally, a systematic review identified a U-shaped dose-response relationship between sleep duration and cognitive impairment [23]. Specifically, self-reported durations of both long and short sleep were linked to the occurrence of MCI [26]. Older individuals sleeping less than 6 h exhibited a 30% increased risk of dementia compared with those with the typical 7 h of sleep [27]. Furthermore, longitudinal studies among middle-aged and older adults have consistently associated both short and long sleep durations with the occurrence of MCI [28–30], reinforcing the nonlinear relationship between nighttime sleep duration and cognitive function. These findings underscore the critical importance of maintaining optimal sleep duration for optimal cognitive function.

Our study identified the optimal nighttime sleep interval for older adults as 6–8 h, with approximately 7 h demonstrating the greatest health benefits. This aligns with previous research indicating that both short (< 6 h/day) and long (> 8 h/day) sleep durations are detrimental to cognitive function [13]. Individuals who deviated from the regular sleep pattern of 6–8 h per night experienced poorer cognitive function [31]. Short sleep durations (0–6 h/night) and long sleep durations (> 9 h/night) were significantly associated with worse cognitive test performance compared with intermediate sleep lengths (6–9 h/night) [32]. In middle-income countries, excessive sleep among older individuals poses a risk factor for certain types of cognitive impairment [30]. Moreover, prolonged sleep has been linked to accelerated frontotemporal gray matter atrophy in older adults, potentially impairing memory [33]. Disruptions in circadian rhythms associated with sleep disorders and cognitive impairment may also contribute to prolonged sleep, leading to irregular circadian rhythms associated with short-term cognitive impairment and long-term brain atrophy [34, 35].

Previous studies have conflicting results compared with our findings. One study reported that participants with short sleep duration (≤ 6.5 h/day) were at higher risk of cognitive decline over time, whereas those with long sleep duration (≥ 8.5 h/day) showed no association [25]. This disparity in findings can be attributed to differences in study design. Our study included a broader age range and considered a wider range of demographic, socioeconomic, behavioral, and health-related confounders compared with previous studies.

### Napping in the middle of the day in the elderly is beneficial to reduce the possibility of MCI

Studies focusing on daytime sleep and cognition in older adults have typically overlooked napping as a primary variable [36, 37]. Therefore, our study sought to investigate the relationship between varying nap durations and MCI. We discovered a nonlinear relationship between midday nap duration and MCI, with the lowest probability of MCI observed among individuals napping for 30–90 min. Specifically, compared with those napping for 30–90 min, older adults without napping habits were more likely to experience MCI, whereas other nap duration groups showed no significant association with MCI.

Moderate napping is linked with better cognition and may play a crucial role in optimizing cognitive performance among older adults. Some studies suggested that cognitive function is better among moderate nappers (30–90 min), whereas non-nappers (0 min) and prolonged nappers (> 90 min) exhibit lower overall cognitive scores [16]. This study also found that older adults with a napping duration of less than 30 min (OR = 0.73, 95% CI: 0.56–0.95) exhibited a lower probability of MCI compared to those without a napping habit.This is consistent with findings indicating a lower prevalence of cognitive impairment among individuals taking short naps (< 30 min) compared with non-nappers and long nappers (≥ 30 min) [38, 39].

Previous research has yielded inconsistent conclusions regarding the effects of short daytime naps (< 30 min) on cognitive function in the elderly.A longitudinal study in Japan revealed that short daytime naps (< 30 min) were more beneficial for cognitive function [38]. Similarly, findings from the Chinese elderly population indicated a positive correlation between napping and enhanced cognition [40]. Conversely, a US cross-sectional study reported that men napping for < 30 min/day were less likely to develop cognitive impairment compared with those napping for longer durations (≥ 120 min/day), yet no association was found between napping for more than 30 min and cognitive impairment relative to non-nappers [41].

These divergent conclusions stem from differences in study design, including variations in participants, assessment tools, definitions of habitual nappers, and selection of covariates. Additionally, discrepancies may arise from variations in sleep data collection, such as timing and duration of naps, as well as differences in circadian rhythms and nighttime sleep patterns [42, 43].

### Limitations

The main limitation of this study is that the sleep time was self-reported. This self-reporting method may affect the accuracy and reliability of the data, particularly in the MCI group, where the risk of bias is especially high. Participants in the MCI group may have greater errors or inaccuracies in reporting their sleep time, which could impact the credibility of the study results. Second, the assessment of sleep duration was simplistic, lacking specialized sleep-related scales and comprehensive evaluation dimensions. Although some correlations were observed, the relationship between sleep and nuanced cognitive function requires further investigation. Third, this study is limited by its cross-sectional design, which precludes the establishment of a causal relationship between sleep and cognitive impairment in chronological order. Additionally, the subjective measurement of sleep in this study falls short of objective measurement standards. Therefore, future research can benefit from conducting cohort studies and incorporating objective measurement indicators to facilitate more in-depth analyses.

## Conclusion

After controlling for potential confounding variables, a nonlinear relationship was observed between both nighttime sleep duration and midday nap duration with MCI among the elderly, presenting a “U”-shaped relationship. The probability of depression was lower among older adults with a nap duration of approximately 60 min. Additionally, the optimal nighttime sleep duration falls within the 6–8 h range, with around 7 h of nighttime sleep providing the greatest health benefit in terms of cognitive function.

## Data Availability

Data is publicly available. See: https://charls.charlsdata.com/pages/Data/2020-charls-wave5/zh-cn.html. If you can’t open the URL, Please contact the corresponding author for data requests.

## Abbreviations

AD: Alzheimer’s Disease
AACD: Aging-Associated Cognitive Decline
HRS: Health and Retirement Study
VIF: Variance Inflation Factor
MCI: Mild Cognitive Impairment
CHARLS: China Health and Retirement Longitudinal Study
OR: Odds Ratio
CI: Confidence Interval
